# Fighting time: the critical importance of pre-TAVR mortality risk prediction

**DOI:** 10.1007/s00392-025-02698-1

**Published:** 2025-06-23

**Authors:** Jasmin Shamekhi, Marius Ebert, Angelina Lorek, Irina Eckardt, Baravan Al-Kassou, Mustafa Mousa Basha, Marcel Weber, Miriam Silaschi, Farhad Bakhtiary, Georg Nickenig, Sebastian Zimmer

**Affiliations:** 1 Heart Center Bonn, Department of Medicine II, University Hospital Bonn, Venusberg-Campus 1, 53127 Bonn, Germany; 2 Heart Center Bonn, Cardiac Surgery, University Hospital Bonn, Bonn, Germany

**Keywords:** Aortic valve stenosis, TAVR, Transcatheter aortic valve replacement, Mortality rate, Waiting time for TAVR, Risk stratification, IMPACT score

## Abstract

**Background:**

Symptomatic severe aortic valve stenosis (AS) is a life-threatening condition requiring prompt medical attention. While transcatheter aortic valve replacement (TAVR) is an effective treatment, current scheduling practices often do not account for individual patient risk profiles due to limited data on mortality rates during the waiting period and a lack of viable risk assessment. Consequently, non-prioritized wait times may be unacceptably long for high-risk patient populations.

**Objective:**

This study aimed to evaluate the mortality rate of patients with symptomatic severe AS awaiting TAVR and identify pragmatic clinical risk predictors during this period.

**Methods:**

Between January 2019 and December 2023, 2,454 patients with symptomatic severe AS, were scheduled for TAVR after an interdisciplinary Heart Team discussion at the Heart Center Bonn. Mortality during the waiting period was assessed, and the characteristics of survivors (patients who underwent TAVR) were compared to non-survivors (patients who died before the procedure).

**Results:**

The median waiting time for TAVR was 41 days. A total of 105 (4.3%) patients died during the waiting period, with a median time to death of 29 days. By comparison, 30 day post-TAVR mortality, including the intervention, was 1.7%. Multivariate regression analysis identified independent predictors of pre-TAVR mortality including reduced left ventricular ejection fraction, decreased estimated glomerular filtration rate, mitral regurgitation, tricuspid regurgitation, and advanced heart failure symptoms. An IMPACT score, incorporating these parameters, strongly predicted outcome with a hazard ratio for mortality of 2.1 greatly outperforming both EuroSCORE II and STS-PROM. The IMPACT score of ≥ 5 identified high-risk patients with a pre-TAVR mortality rate of 12.6%.

**Conclusion:**

The mortality rate for patients with symptomatic severe AS awaiting TAVR is unacceptably high. Utilizing the IMPACT score could enable precise risk stratification, identifying patients who require urgent or prioritized intervention to improve outcomes.

**Graphical abstract:**

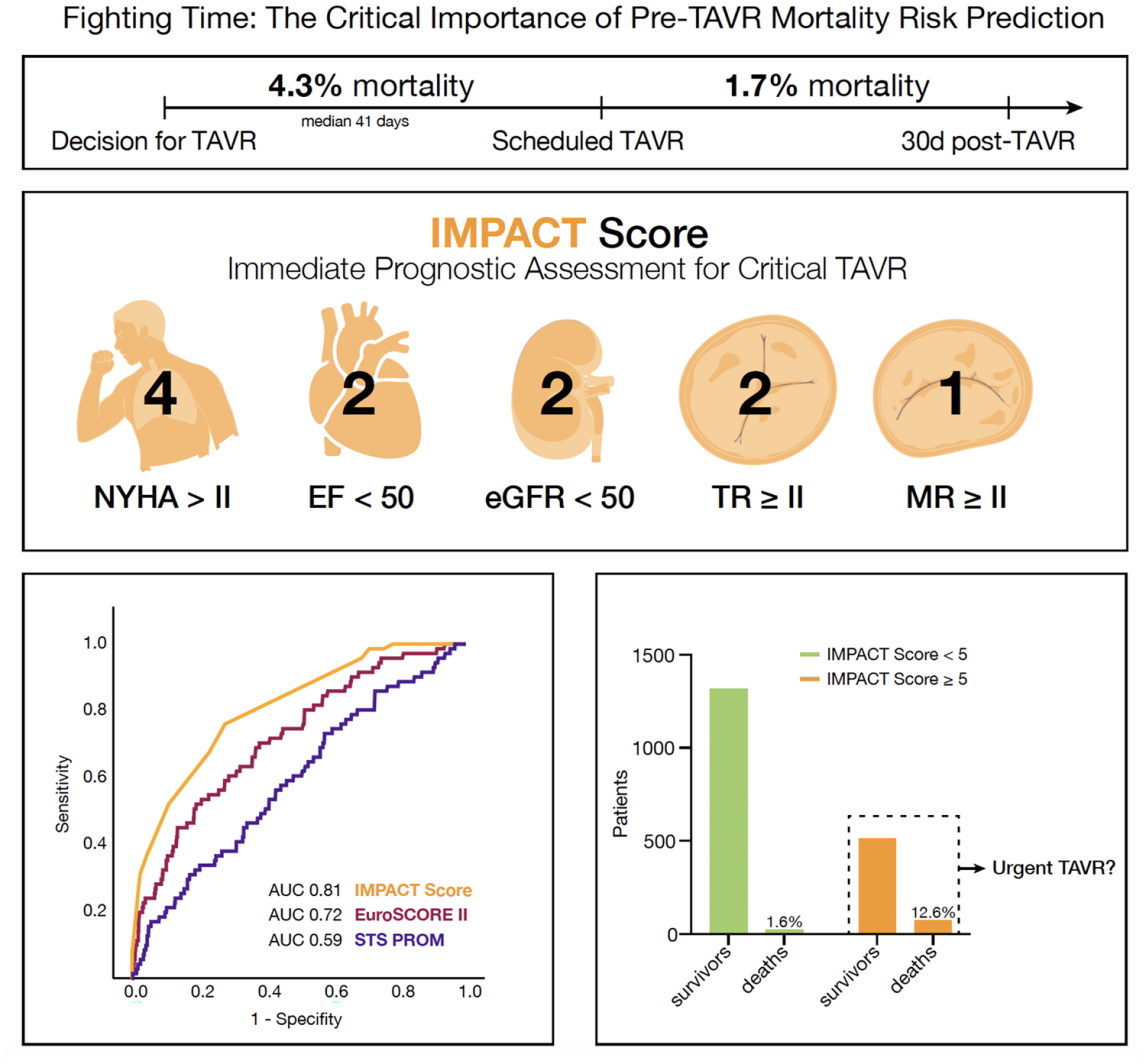

## Introduction

Aortic stenosis (AS) is one of the most common heart valve diseases among the elderly, and its prevalence is expected to increase due to aging populations. This trend poses significant socio-economic and healthcare challenges [1] The prognosis of untreated patients with severe AS is very poor with mortality rates of approximately 25% at 1 year and 50% at 2 years following symptom onset [2].

The only definitive treatment for severe AS is valve replacement, achieved through either surgical aortic valve replacement (SAVR) or transcatheter aortic valve replacement (TAVR) [3–8]. Over the past years, TAVR has emerged as an established treatment option in patients with severe AS across all stages of surgical risk and in 2023, more than 30.000 patients underwent TAVR in Europe. However, the waiting lists for interventional valve replacement are long. Most European countries have wait times of more than 3 months [9]. In France, for example, the mean time to TAVR is 144.2 ± 83.87 days [10] and the UK TAVI Survey from 2019 reported an average time to TAVR of more than 155 days [11]. These times contrast with risks and complications attributed to severe AS and delays in performing TAVR after diagnosis may exacerbate AS progression and heighten the risk of adverse outcomes including the risk of mortality [3].

These considerations compelled us to analyze pre-TAVR mortality, quantify the risks associated with delaying the procedure, and identify clinical characteristics associated with increased risk during the waiting period.

## Methods

### Study design and patient population

We included a total of 2454 patients, who were diagnosed with symptomatic severe AS, and scheduled for TAVR after interdisciplinary discussion by the local, institutional Heart Team. Data acquisition was approved by the local ethics committee and written informed consent was obtained from all patients during hospital admission.

All patients underwent a comprehensive evaluation including pre-interventional transthoracic and transesophageal echocardiography (including three-dimensional measurements), coronary angiography, angiological examination, and pulmonary function test. Details about patient screening and aortic valve evaluation have been described previously [8, 12, 13].

Immediately after diagnosis and Heart Team discussion, patients were scheduled for TAVR at the earliest possible date. Additionally, all patients were treated in accordance with ESC guidelines, including an optimal medical therapy in patients with heart failure symptoms or cardiac decompensation, rhythm or rate control in patients with cardiac arrhythmias or revascularization in patients with concomitant coronary artery disease (CAD).

Within the study cohort, we assessed the mortality rate in the time between determining the indication for TAVR and the procedure itself and compared baseline clinical characteristics and echocardiographic parameters between survivors and non-survivors. We further assessed risk factors associated with pre-TAVR mortality, aiming to identify key clinical, demographic, and procedural variables that contribute to increased risk during the waiting period. These factors were systematically analyzed to better understand their relative impact on mortality and develop a more precise and practical risk assessment tool: Immediate Prognostic Assessment for Critical TAVR (IMPACT) score.

### IMPACT score

We analyzed all baseline parameters to identify their potential associations with pre-TAVR mortality using a univariate Cox regression analysis. Next, all parameters that demonstrated significant associations in the univariate analysis were included in a subsequent multivariable regression analysis. The parameters that remained statistically significant in the multivariable analysis were deemed independently associated with pre-TAVR mortality and were selected for inclusion in the IMPACT score. To ensure accurate representation of their relative influence, the regression coefficient (β value) from the multivariable analysis was used to assign weights to each parameter within the score. This weighting method ensured that each parameter's contribution to the overall score reflected its proportional impact on the risk of mortality.

### Statistical analysis

Data are presented as the mean ± standard deviation, if normally distributed, or as the median and an interquartile range (IQR) (quartile 1/quartile 3), if not normally distributed. Continuous variables were tested for having a normal distribution with the use of the Kolmogorov–Smirnov test. Categorical variables are given as frequencies and percentages. For continuous variables, a Student’s *t* test or a Mann–Whitney *U* test, as appropriate, was performed for comparing two groups. When comparing more than two groups, ANOVA or the Kruskal–Wallis test was used. Spearman’s correlation coefficients were used to establish associations. The χ^2^ test was used for analysis of categorical variables. We performed a multivariate Cox regression analysis, which included univariate predictors with a *p* value < 0.05, to assess independent predictors for pre-TAVR mortality.

To create the new risk score, we converted the regression coefficients of all independent predictors for pre-TAVR mortality into a scoring system. Finally, we used ROC (receiver operating characteristic) curve and Youden Index to identify the optimal cutoff points for classifying patients into distinct risk groups. This method allowed us to maximize the combined sensitivity and specificity, ensuring an effective balance between identifying high-risk patients and minimizing false positives. Based on this cutoff value, we stratified the population into a low- (IMPACT score < 5) and a high-risk (IMPACT score ≥ 5) group, facilitating a more nuanced understanding of risk distribution.

Survival according to the IMPACT score was determined with use of the Kaplan–Meier method. The log-rank test was used to determine statistical differences in terms of survival.

ROC curve analysis was used to evaluate the discrimination ability of several risk assessment tools. The area under the curve (AUC) was calculated for each tool to quantify their overall performance, with higher AUC values indicating superior discrimination between patients at high and low risk of adverse outcomes.

Statistical significance was assumed when the null hypothesis could be rejected at *p* < 0.05. Statistical analyses were conducted with SPSS Statistics version 29.0.0.0 (IBM Corporation, Somers, NY, USA). The investigators initiated the study, had full access to the data, and wrote the manuscript. All authors vouch for the data and its analysis.

## Results

### Study population and outcome

A total of 2,454 patients were included in the study. The mean age was 80.8 years (± 6.5 years) and 57.9% were male. The cohort had an intermediate operative risk, reflected by a mean EuroSCORE II of 5.5 (± 7.0) and a mean STS-PROM of 5.9 (± 5.4).

**Fig. 1:**
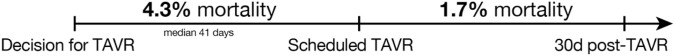
Mortality rate before and after scheduled TAVR. The median waiting time from indication to TAVR was 41 days. From the overall cohort, 4.3% patients died during the waiting time, whereas the 30-day mortality rate after TAVR was 1.7%

The median waiting time from indication to TAVR was 41 days (IQR 31–62). During the waiting period for TAVR, 105 patients (4.3%) died, while 2,349 underwent the procedure (Fig. 1). The median time to death for non-survivors was 29 days (IQR 10–49). Cardiovascular disease was the leading cause of death (70 patients), followed by infectious diseases (12 patients), neurological disorders (8 patients), cancer (2 patients), and unknown causes (13 patients). In contrast, the 30-day all-cause mortality rate after TAVR was significantly lower at 1.7%, with deaths primarily related to procedural complications (Fig. 1).

### Baseline clinical characteristics and echocardiographic parameters

Baseline characteristics and echocardiographic parameters are detailed in Table 1. Patients who died during the waiting period exhibited significantly higher surgical risk scores (EuroSCORE II: 10.9 ± 9.8 vs. 5.3 ± 6.8, *p* < 0.001; STS-PROM: 7.5 ± 6.3 vs. 5.9 ± 5.4, *p* = 0.015) and were more likely to have undergone prior cardiac surgery (19.8% vs. 12.7%, *p* = 0.04). Non-survivors also had higher rates of comorbidities, including chronic kidney disease (29.4% vs. 10.0%, *p* < 0.001), chronic obstructive pulmonary disease (COPD) (31.0% vs 15.8%, *p* = 0.001), and atrial fibrillation (60.0% vs. 44.0%, *p* = 0.04), though cardiovascular risk factors, such as hypertension, hypercholesterolemia, and diabetes mellitus type II, were comparable between groups.

**Table 1:** Baseline characteristics and echocardiographic parameters

	All patients *n* = 2454	Survivors *n* = 2349	Non-Survivors *n* = 105	*p* value
Age, years (SD)	80.8 (± 6.5)	80.9 (± 6.5)	80.1 (± 6.9)	0.41
Male, n (%)	1407 (57.5)	1336 (57.1)	71 (67.6%)	0.054
Height, cm (SD)	169.6 (± 8.9)	169.6 (± 9.0)	170.0 (± 7.9)	0.19
Weight, kg (SD)	77.0 (± 16.1)	76.9 (± 16.0)	81.5 (± 17.4)	**0.03**
NYHA class, *n* (%)				** < 0.001**
0	30 (1.3)	29 (1.4)	1 (1.0)	
I	102 (4.6)	99 (4.6)	3 (3.0)	
II	555 (24.9)	554 (26.0)	1 (1.0)	
III	1398 (62.7)	1308 (61.4)	90 (89.1)	
IV	146 (6.5)	140 (6.6)	6 (5.9)	
COPD, *n* (%)	305 (8.5)	279 (15.8)	26 (31.0)	**0.001**
PAD, *n* (%)	1086 (46.2)	1066 (46.1)	20 (51.3)	0.52
Previous Pacemaker, *n* (%)	296 (12.5)	292 (12.5)	4 (10.0)	0.63
Atrial fibrillation, *n* (%)	1051 (44.3)	1027 (44.0)	24 (60.0)	**0.04**
Oral anticoagulation, *n* (%)	1142 (48.2)	1118 (48.0)	24 (60.0)	0.13
Previous stroke, *n* (%)	275 (11.7)	269 (11.6)	6 (15.0)	0.50
Hypertension, *n* (%)	2200 (90.5)	2110 (90.6)	90 (89.1)	0.62
Diabetes mellitus II, *n* (%)	770 (31.7)	737 (31.6)	33 (32.7)	0.83
Hypercholesterinemia, *n* (%)	1706 (70.2)	1632 (70.1)	74 (73.3)	0.49
Chronic kidney disease, *n* (%)	264 (10.8)	234 (10.0)	30 (29.4)	** < 0.001**
Previous cardiac surgery, *n* (%)	316 (13.0)	296 (12.7)	20 (19.8)	**0.04**
EuroSCORE II, % (SD)	5.5 (± 7.0)	5.3 (± 6.8)	10.9 (± 9.8)	** < 0.001**
STS-PROM, % (SD)	5.9 (± 5.4)	5.9 (± 5.4)	7.5 (± 6.3)	**0.015**
Ejection fraction, % (SD)	54.4 (± 11.2)	54.7 (± 10.9)	47.1 (± 13.3)	** < 0.001**
Aortic regurgitation ≥ II, *n* (%)	270 (11.9)	245 (11.3)	25 (25.3)	** < 0.001**
Mitral regurgitation ≥ II, *n* (%)	451 (19.2)	395 (17.6)	56 (56.0)	** < 0.001**
Tricuspid regurgitation ≥ II, *n* (%)	331 (14.3)	283 (12.8)	48 (48.0)	** < 0.001**
Coronary artery disease, *n* (%)	1618 (66.6)	1545 (66.4)	73 (72.3)	0.22
Prior myocardial infarction, *n* (%)	216 (12.9)	207 (12.5)	9 (52.9)	< 0.001
AV MPG, mmHg (SD)	39.3 (± 15.3)	39.4 (± 15.2)	35.6 (± 16.6)	**0.008**
AV Vmax, m/s (SD)	3.7 (± 0.8)	3.7 (± 0.8)	3.8 (± 0.8)	0.06
AVA, cm^2^ (SD)	0.8 (± 0.2)	0.8 (± 0.2)	0.8 (± 0.2)	0.30
sPAP, mmHg (SD)	38.0 (± 14.3)	37.8 (± 14.2)	41.7 (± 15.0)	**0.007**
NT-proBNP, pg/ml (IQR)	1389 (550/3642)	1357 (544/3523)	7862 (1911/24088)	** < 0.001**
Creatinine, mg/dl (IQR)	1.08 (0.87/1.39)	1.08 (0.87/1.38)	1.6 (1.01/2.2)	** < 0.001**
Troponin, ng/l (IQR)	26.8 (17.1/47.4)	26.2 (17.0/45.7)	86.0 (40.0/202.7)	** < 0.001**

Values in bold indicate statistical significance (*p* < 0.05)

Echocardiographic analysis revealed that non-survivors had significantly lower left ventricular ejection fraction (EF) (47.1 (± 13.3) vs. 54.7 (± 10.9), *p* < 0.001) and a higher prevalence of concomitant valvular diseases (mitral regurgitation ≥ moderate: 56.0% vs. 17.6%, *p* < 0.001; tricuspid regurgitation ≥ moderate: 48.0% vs. 12.8%, *p* < 0.001). Systolic pulmonary artery pressure (sPAP) was also elevated in non-survivors (41.7 (± 15.0) vs. 37.8 (± 14.2), *p* = 0.007), with higher NT-pro-BNP levels (7862 pg/ml [IQR 1911–24,088] vs. 1357 pg/ml [IQR 544–3523], *p* < 0.001).

Interestingly, aortic valve area (AVA) and peak aortic jet velocity (Vmax) were similar between groups, but non-survivors had significantly lower mean pressure gradients (35.6 ± 16.6 mmHg vs. 39.4 ± 15.2 mmHg, *p* = 0.008), likely due to a higher prevalence of low-flow, low-gradient aortic stenosis.

### IMPACT score

Univariable Cox regression analysis identified several factors associated with pre-TAVR mortality, including NYHA class > II, COPD, chronic kidney disease, defined as an estimated glomerular filtration rate < 50 ml/min/1,73 m^2^, atrial fibrillation, pulmonary hypertension, reduced ejection fraction, and moderate or severe mitral and tricuspid regurgitation (Table 2). Multivariable analysis confirmed significant associations for reduced left ventricular ejection fraction, decreased estimated glomerular filtration rate, mitral regurgitation, tricuspid regurgitation, and advanced heart failure symptoms (Table 2).

**Table 2:** Cox Regression analysis for association with pre-TAVR mortality

	Univariate analysis HR (95% CI)	*p*-value	Multivariate analysis HR (95% CI)	*p* value
NYHA class > II	28.3 (3.92–204.2)	** < 0.001**	20.3 (2.8–147.1)	**0.003**
COPD	2.08 (1.18–3.68)	**0.011**	0.66 (0.31–1.39)	0.275
Chronic kidney disease	5.05 (2.97–8.58)	** < 0.001**	3.07 (1.76–5.34)	** < 0.001**
Atrial fibrillation	4.94 (1.05–23.1)	**0.04**	2.20 (0.43–11.21)	0.34
Pulmonary hypertension	1.02 (1.00–1.04)	**0.01**	0.99 (0.97–1.01)	0.69
Ejection fraction < 50%	3.73 (2.17–6.41)	** < 0.001**	3.09 (1.79–5.35)	** < 0.001**
MR ≥ moderate	5.85 (3.49–9.83)	** < 0.001**	2.02 (1.10–3.72)	**0.02**
TR ≥ moderate	8.23 (4.09–13.83)	** < 0.001**	4.02 (2.19–7.36)	** < 0.001**
NT-proBNP	2.61 (0.86–7.88)	0.08	–	–

Values in bold indicate statistical significance (*p* < 0.05)

**Fig. 2:**
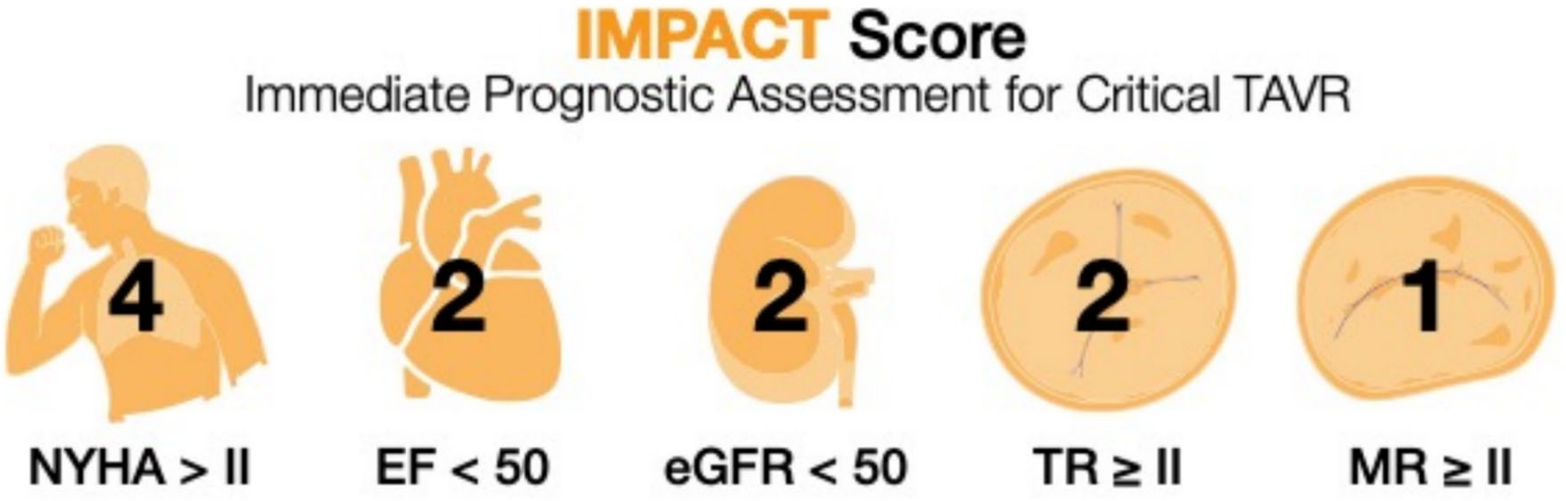
Composition of the Immediate Prognostic Assessment for Critical TAVR Score (IMPACT). The IMPACT score consists of five parameters including a reduced left ventricular ejection fraction, a decreased estimated glomerular filtration rate, mitral regurgitation, tricuspid regurgitation, and advanced heart failure symptoms

**Fig. 3:**
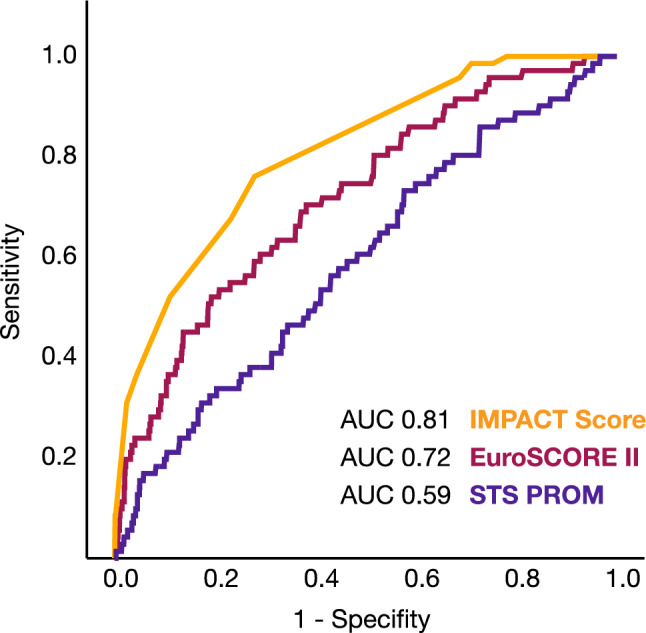
ROC analysis comparing different scoring systems for pre-TAVR mortality. The IMPACT score demonstrated strong predictive accuracy, with an area under the curve (AUC) of 0.81 for pre-TAVR mortality. It outperformed both the EuroSCORE II and STS-PROM in mortality prediction

**Fig. 4:**
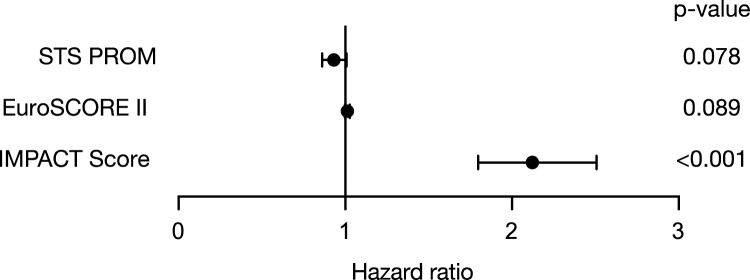
Forest plot comparing the hazard ratio predicted by different scoring systems for pre-TAVR mortality. The IMPACT score predicted a hazard ratio for mortality of 2.122 (95% CI: 1.797–2.507)

The IMPACT score, derived from these parameters (Fig. 2), demonstrated strong predictive accuracy, with an area under the curve (AUC) of 0.81 for pre-TAVR mortality (Fig. 3). It outperformed both EuroSCORE II and STS-PROM in mortality prediction. The score predicted a hazard ratio for mortality of 2.122 (95% CI: 1.797–2.507) (Fig. 4).

**Fig. 5:**
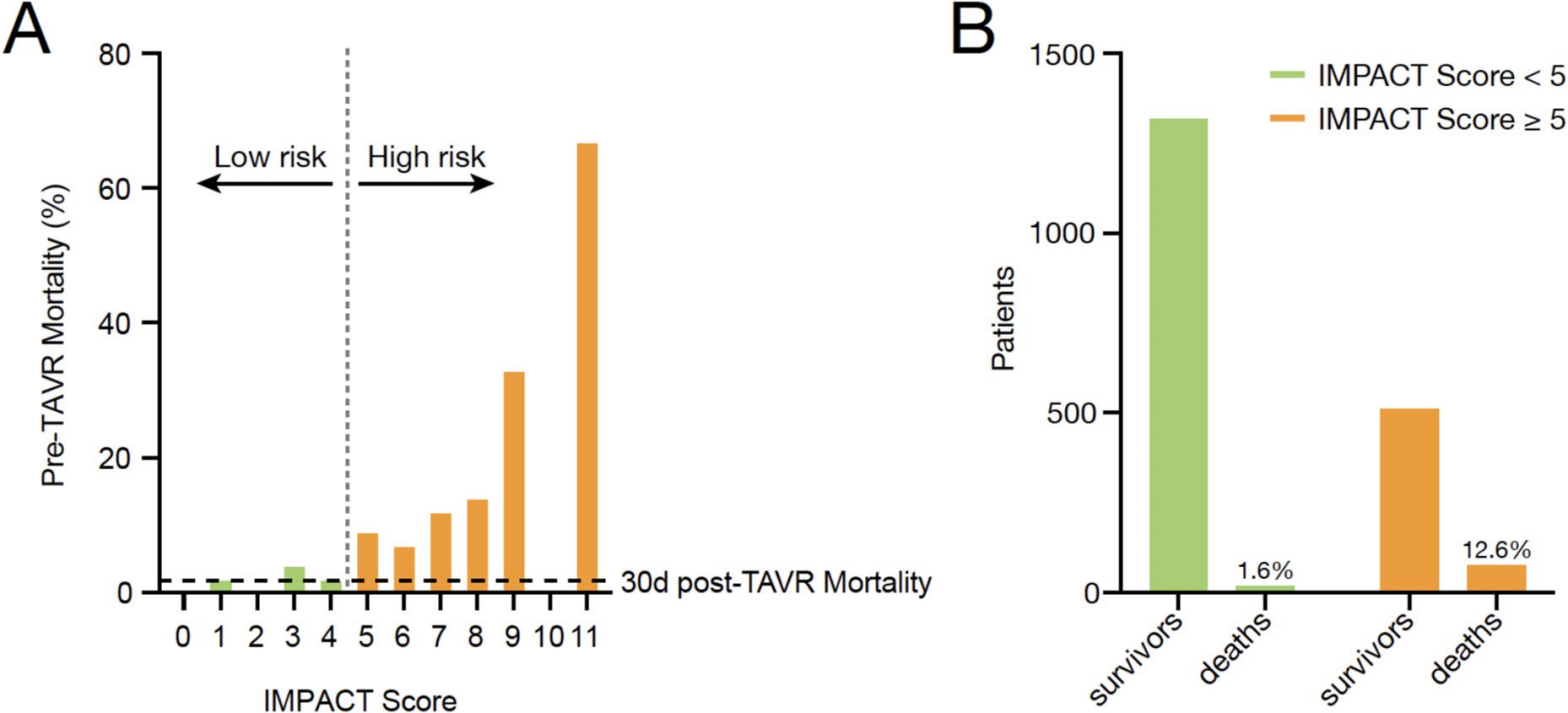
**A** Observed pre-TAVR mortality of patients for each point value of the IMPACT score. In the low-risk group, the pre-TAVR mortality rate was similar to the baseline 30-day post-TAVR mortality rate of 1.7%. **B** Number of patients who survived or died, split by low and high IMPACT score. Patients with an IMPACT score ≥ 5 had significantly higher pre-TAVR mortality rates of 12.6%

**Fig. 6:**
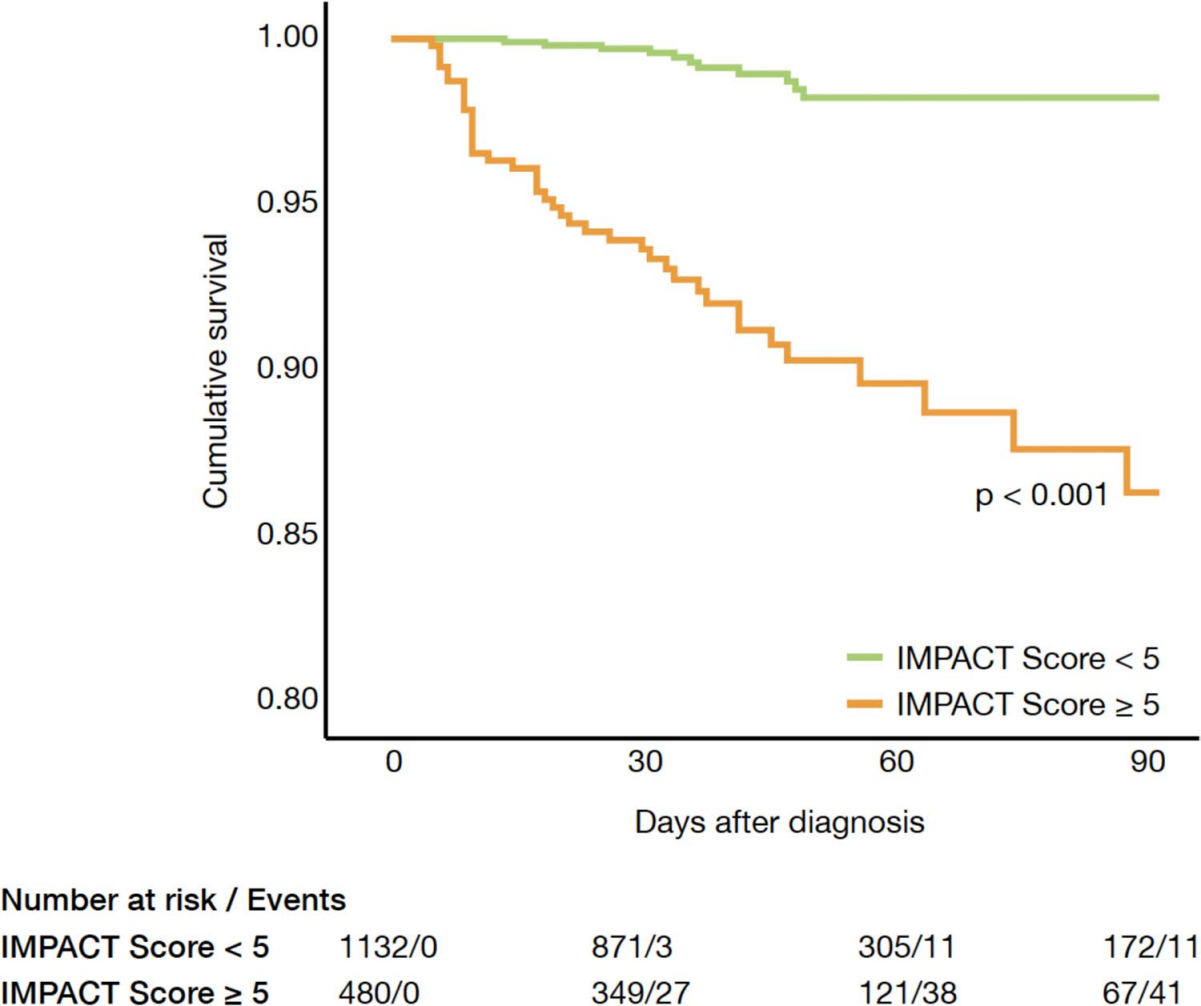
Pre-TAVR cumulative survival in low and high IMPACT score cohort. Patients with an IMPACT score ≥ 5 had significantly higher pre-TAVR mortality rates compared to patients with an IMPACT score < 5

According to the receiver operating characteristics (ROC) curve analysis and the Youden Index, patients with an IMPACT score < 5 were defined as low-risk patients, whereas an IMPACT score ≥ 5 was identified to characterize high-risk patients for pre-TAVR mortality. In the low-risk group, the pre-TAVR mortality rate was similar to the baseline 30 day post-TAVR mortality rate of 1.7% (Fig. 5A). In contrast, patients with an IMPACT score ≥ 5 (586 patients; 23.9% of the cohort) had significantly higher pre-TAVR mortality rates of 12.6% (Figs. 5B and 6).

## Discussion

This is the first study to characterize individual risk in patients with symptomatic severe AS awaiting TAVR. Among these patients, the mortality rate was 4.3% during an average waiting time of 41 days. Notably, the median time from establishing the treatment indication to death was just 29 days. The newly developed IMPACT score effectively identified high-risk patients at greatest risk of mortality during the waiting period, highlighting its potential to guide prioritization and improve patient care.

Several studies have investigated the impact of pre-TAVR waiting time on post-procedural outcome [10, 14, 15]However, there are only very limited data evaluating the pre-TAVR mortality. *Albassam *et al*.* investigated the association between wait times for TAVR and mortality or hospitalization related to heart failure while on wait list. The authors described a mortality of 5.2% during a median wait time of 84 days in a cohort of 8098 referrals for TAVR in Ontario, Canada [16]. In contrast to our study, the outlined analysis included patients between 2012 and 2018 with a high operative risk, while our overall cohort represented a more contemporary, intermediate risk population.

Our observed mortality rate of 4.3% in patients awaiting TAVR was more than twofold higher than the 30-day all-cause mortality rate after TAVR (1.7%). This emphasizes the severity of symptomatic AS and that the waiting period before treatment is a critical window where patients are at significant risk of death. Additionally, prolonged TAVR has socio-economic relevance as earlier data have demonstrated an association between longer wait time and increased health care costs [17]. Simulation models including a mock population of 50,000 individuals with complete follow-up from referral to TAVR therefore propose a wait time of less than 3 weeks for high-risk patients [18].

The waiting time for TAVR varies greatly between different countries and geographic regions. *Rosseel *et al*.* described a waiting time of > 3 months in most European countries including France, Belgium, Luxembourg, Denmark, Finland, the United Kingdom, and the Republic of Ireland. Germany, Austria, and Switzerland were the only regions with a waiting period < 3 months [9]. The median waiting time to TAVR in Sweden has been shown to be 53 days [15] and even 148 days in Australia [14]. Available data from the USA reported a median waiting time from AVR recommendation to intervention (including SAVR and TAVR) of 2.9 weeks in an observational period between 2008 and 2012 [19]. In our study, the median time from treatment indication and scheduling TAVR to death was 29 days. Interestingly, *Stehli *et al*.* described a significantly longer waiting period in women compared to men in a total of 407 patients undergoing TAVR in Australia [14]. In our cohort, waiting periods were similar in both men and women. The pre-TAVR mortality was also comparable (*p* = 0.71).

Waiting times before TAVR are influenced by several interconnected factors. These include logistical elements, such as hospital workflow and resource availability; systemic issues, such as the efficiency of the healthcare system, geographic location, referral processes, and patient compliance; and clinical considerations, such as the patient's risk profile, comorbidities, and overall stability. These factors often compete with one another, making it impractical to universally reduce pre-TAVR waiting times for all patients.

Furthermore, while the development of severe AS may take years, this does not automatically classify aortic valve replacement as an elective procedure for every patient. Instead, individualized risk assessments should be considered to optimize the timing and planning of TAVR.

The introduced IMPACT score is a novel risk stratification tool that relies on five straightforward and clinically relevant parameters: NYHA classification, renal function, left ventricular ejection fraction, and the presence of a concomitant moderate-to-severe mitral and tricuspid valve regurgitation. The variables included in the IMPACT score were chosen through a structured, two-step statistical approach. A set of clinically relevant candidate variables was initially evaluated using univariable logistic regression analyses to assess their association with mortality. Variables reaching statistical significance (*p* < 0.05) in the univariable analysis were subsequently included in a multivariable logistic regression model. Only predictors that remained independently associated with mortality in the multivariable model were selected for inclusion in the final score. The β-coefficients derived from the multivariable analysis were used to assign points to each predictor. To simplify the model and facilitate clinical applicability, β-coefficients were scaled and rounded to the nearest integer, resulting in a straightforward additive scoring system. The predictive performance of the IMPACT score was assessed using receiver operating characteristic (ROC) curve analysis, and the area under the curve (AUC) was calculated to evaluate discrimination. The IMPACT score effectively identifies high-risk patients at an elevated likelihood of adverse outcomes during the waiting period for TAVR. Importantly, the IMPACT score outperformed well-established risk assessment tools, such as the EuroSCORE II and STS-PROM, in accurately predicting mortality, demonstrating its superior prognostic capabilities. What sets the IMPACT score apart is its simplicity and practicality as it uses routinely collected clinical variables, making it easily implementable in daily practice without the need for completing lengthy and complicated questionnaires. By providing clinicians with a reliable tool for early risk stratification, the IMPACT score allows for better prioritization of high-risk patients, facilitating timely intervention, personalized care plans, and closer monitoring during the critical waiting period.

Ultimately, our study highlights the critical impact of pre-treatment waiting times for patients with severe AS. However, whether urgent TAVR can improve outcomes, particularly in high-risk patients, remains speculative. To address this question and to validate the IMPACT score in a larger multicenter cohort, a prospective randomized trial is warranted.

### Study limitations

A limitation of the study is the sample size including only a small number of patients in the group of the non-survivors. Furthermore, the monocentric study design may hamper an extrapolation of the data. Due to the retrospective study design, only mortality outcomes were available for analysis. Consequently, the predictive performance of the IMPACT score for other clinical endpoints could not be evaluated. Another limitation of our study is that no alternative predictor weightings or formal sensitivity analyses were performed to assess the robustness of the model. Future studies are warranted to validate the IMPACT score in larger, independent, and multicenter cohorts. Additionally, we only included patients with severe aortic valve stenosis and recommendation for TAVR; patients who were scheduled for SAVR were not included.

## Conclusion

The mortality rate of patients with severe symptomatic AS and the indication for treatment (TAVR) is 4.3% during a median waiting period of 41 days. Our study results indicate that a shorter waiting interval from determining the indication for TAVR and the actual procedure is needed in selected high-risk patients. The introduced IMPACT score is able to identify high-risk patients for an adverse outcome during the waiting period for TAVR. Patients with an IMPACT score ≥ 5 points are at markedly increased risk and can perhaps benefit from prioritized or urgent TAVR to reduce mortality.

## Abbreviations

TAVR: Transcatheter aortic valve replacement
AS: Aortic valve stenosis
IQR: Interquartile range
COPD: Chronic obstructive pulmonary disease
LVEF: Left ventricular ejection fraction
SAVR: Surgical aortic valve replacement
CAD: Coronary artery disease
IMPACT score: Immediate Prognostic Assessment for Critical TAVR Score
sPAP: Systolic pulmonary artery pressure
AVA: Aortic valve area
AUC: Area under the curve
ROC: Receiver operating characteristics
